# Expression and role of nicotinic acetylcholine receptors during midbrain dopaminergic neuron differentiation from human induced pluripotent stem cells

**DOI:** 10.1002/npr2.12361

**Published:** 2023-06-27

**Authors:** Takeshi Kato, Kaneyasu Nishimura, Masahiro Hirao, Shun Shimohama, Kazuyuki Takata

**Affiliations:** ^1^ Joint Research Laboratory, Division of Integrated Pharmaceutical Science Kyoto Pharmaceutical University Kyoto Japan; ^2^ Laboratory of Functional Brain Circuit Construction, Graduate School of Brain Science Doshisha University Kyotanabe Japan; ^3^ Department of Neurology, School of Medicine Sapporo Medical University Sapporo Japan; ^4^ Jiseikai Dementia Center Itabashi Japan; ^5^ Jiseikai Nerima Takanodai Hospital Nerima Japan

**Keywords:** dopaminergic neurons, human induced pluripotent stem cells, *LMO3*, nicotinic acetylcholine receptors

## Abstract

**Aim:**

Nicotinic acetylcholine receptors (nAChRs) expressed in midbrain dopaminergic (mDA) neurons modulate mDA neuronal activity. However, their expression patterns and functional roles during mDA neuronal development remain unknown. Here, we profiled the expression and function of nAChR subtypes during mDA neuron differentiation from human induced pluripotent stem cells (hiPSCs).

**Methods:**

Midbrain dopaminergic neurons were differentiated from hiPSCs using a recently developed proprietary method that replicates midbrain development. The expression patterns of developmental marker proteins were monitored during mDA neuronal differentiation using immunohistochemical analysis. Gene expression of nAChR subtypes was analyzed by reverse transcription polymerase chain reaction. Pharmacological nAChR agonists and antagonists were used to reveal the role of the α6 nAChR subunit in the differentiation of mDA neurons from hiPSCs.

**Results:**

*CHRNA4* expression was detected at the mDA neural progenitor stage, whereas *CHRNA6* expression began during the mDA neuronal stage. *CHRNA7* was expressed throughout the differentiation process, including in the undifferentiated hiPSCs. We also found that *LMO3*, a gene expressed in a subset of substantia nigra pars compacta (SNC) DA neurons in the midbrain, showed increased expression following nicotine treatment in a concentration‐dependent manner. Additionally, 5‐iodo A85380, a selective α6 nAChR agonist, also increased *LMO3* expression in hiPSC‐derived mDA neurons, and this increase was suppressed by simultaneous treatment with bPiDi, a selective α6 nAChR antagonist.

**Conclusion:**

Our findings suggest that stimulating the α6 nAChR subunit on hiPSC‐derived mDA neurons may induce neuronal maturation that is biased toward SNC DA neurons.

## Abstract 2

Here, we profiled the expression and function of nicotinic acetylcholine receptor (nAChR) subtypes during midbrain dopaminergic (mDA) neuron differentiation from human induced pluripotent stem cells (hiPSCs). CHRNA4 expression was detected at the mDA neural progenitor stage, whereas CHRNA6 expression began during the mDA neuronal stage. CHRNA7 was expressed throughout the differentiation process, including in the undifferentiated hiPSCs. We also found that LMO3, a gene expressed in a subset of substantia nigra pars compacta DA neurons in the midbrain, showed increased expression following nicotine treatment in a concentration‐dependent manner. Additionally, 5‐iodo A85380, a selective a6 nAChR agonist, also increased LMO3 expression in hiPSC‐derived mDA neurons, and this increase was suppressed by simultaneous treatment with bPiDi, a selective a6 nAChR antagonist.

## Graphical abstract

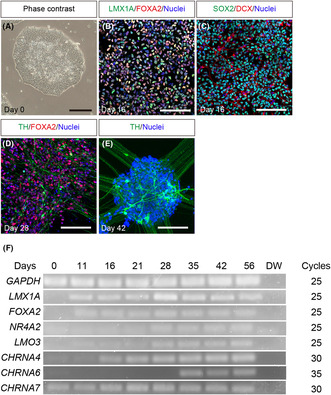

## 1 INTRODUCTION

Nicotinic acetylcholine receptors (nAChRs) are ligand‐gated cation channels that are distributed throughout entire organs, including the central nervous system.^1^ nAChRs are pentameric compositions from either 10 α‐subunits (α1–10) or four β‐subunits (β1–4), which results in varying properties and functional diversity.^2^ The α4, α6, and α7 nAChR subunits are expressed in dopaminergic (DA) neurons in the midbrain and modulate midbrain DA (mDA) neuronal activity.^3^, ^4^, ^5^, ^6^

Dopaminergic neurons are a major cell population in the ventral midbrain that is involved in voluntary movement, cognition, motivation, and reward. mDA neurons can be anatomically and functionally distinguished into neurons projecting from three main nuclei: the substantia nigra pars compacta (SNC), ventral tegmental area (VTA), and red nucleus.^7^, ^8^, ^9^ SNC DA neurons project to the caudate‐putamen and form the nigrostriatal pathway. Loss of SNC DA neurons is a defining pathological feature of Parkinson's disease (PD) and may contribute to tremor, rigidity, and loss of postural control.^10^ Recently, mDA neurons generated from human pluripotent stem cells (PSCs) have been used as an experimental model for drug discovery and as a source of cell transplantation therapy for PD.^11^, ^12^, ^13^, ^14^ Moreover, the current advantageous aspects are generating either SNC or VTA DA neurons from human PSCs by controlling the signaling functions during the brain developmental process.^15^, ^16^

The LIM‐only protein (LMO) is a LIM domain‐containing protein family that is involved in cell fate determination during embryonic development.^17^ LMO3 is a coregulator that is enriched in the SNC and may contribute to Pitx3‐dependent transcription.^18^ A single‐cell RNA‐sequencing (scRNA‐seq) dataset from the human ventral midbrain during embryonic development revealed that mDA neurons can be divided into three subpopulations—DA0, DA1, and DA2—with the DA2 subpopulation specifically expressing *LMO3*.^19^ Interestingly, the dataset also indicated that some nAChRs were expressed in DA populations during midbrain development, with *CHRNA6* specifically expressed in the DA2 subpopulation, whereas *CHRNA4* and *CHRNA7* were broadly expressed among ventral midbrain neural populations.^19^

Although nAChRs are known to be expressed during mDA neuron differentiation, the timing of this expression and the function of nAChRs are not fully understood. Here, we performed nAChR expression profiling using a model that differentiates human‐induced PSCs (hiPSCs) into mDA neurons. Furthermore, we investigated the role of α6 nAChRs during mDA neuron differentiation from hiPSCs using pharmacological approaches.

## 2 METHODS

### 2.1 hiPSC culture

The hiPSC line 1231A3 was established by Kyoto University, derived from ePBMC® purchased from Cellular Technology Limited (http://www.immunospot.com/), and provided by the RIKEN Bioresource Research Center (Tsukuba, Japan) through the National BioResource Project of the Ministry for Education, Culture, Sports, Science, and Technology/Japan Agency for Medical Research and Development (MEXT/AMED), Japan.^20^ hiPSCs were cultured following a previously described method.^21^

### 2.2 Induction of mDA neurons

Midbrain DA neuron induction was slightly modified from a previously described protocol.^22^ Briefly, the cells were seeded at a density of 500 000 cells/cm^2^ on iMatrix511‐silk (Nippi, Tokyo, Japan)‐coated dish in E8 medium (Thermo Fisher Scientific, Waltham, MA, USA) with 10 μM Y27632 (Selleck, Houston, TX, USA). The cells were differentiated in E6 medium (Thermo Fisher Scientific) supplemented with 1% nonessential amino acids (Fujifilm Wako Chemicals, Osaka, Japan), 1% GlutaMax (Thermo Fisher Scientific), and 0.1 mM 2‐mercaptoethanol (Fujifilm Wako Chemicals). Additionally, the E6 medium was supplemented with 200 nM LDN193189 (Selleck) and 500 nM A83‐01 (Fujifilm Wako Chemicals) from day 0 to 11, 2 μM purmorphamine (Selleck) from day 1 to 16, and 1.5 μM CHIR99021 (Selleck) from day 3 to 11. The medium was gradually changed to neurobasal medium (Thermo Fisher Scientific) with B27 supplement vitamin A minus (Thermo Fisher Scientific) and 1% GlutaMax from day 5 to day 11. From day 11 to day 16, 7.5 μM CHIR99021 was further added to the neurobasal medium. On day 16, the cells were dissociated into single cells, replated at a density of 750 000 cells/cm^2^ on iMatrix511‐silk‐coated dish, and were cultured through day 21 with neurobasal medium containing 10 μM GW3965 (Selleck), 10 μM DAPT (Selleck), 20 ng/mL brain‐derived neurotrophic factor (Peprotech), and 200 μM ascorbic acid (Sigma‐Aldrich, St. Louis, MO, USA). Within 24 h after replating, 10 μM Y27632 was added to the medium. After day 21, 10 ng/mL glial cell‐derived neurotrophic factor (Peprotech), 500 μM dbcAMP (Selleck), and 1 ng/mL transforming growth factor β3 (Peprotech) were added to the medium.

### 2.3 Drug expose

(−)‐Nicotine hydrogen tartrate salt (Sigma‐Aldrich, SML1236), bPiDi (Sigma‐Aldrich, SML0182), and 5‐iodo A85380 dihydrochloride (Abcam, Cambridge, U.K., ab120599) were used. All were dissolved in dimethyl sulfoxide or PBS(−) to prepare a stock concentration of 10 mM.

### 2.4 Immunostaining

Cells were fixed with 4% paraformaldehyde (Nacalai Tesque, Kyoto, Japan) for 20 min at 4°C, then blocked with 5% normal donkey serum (Jackson ImmunoResearch, West Grove, PA, USA) in 0.1 M phosphate‐buffered saline containing 0.1% Triton‐X (Nacalai Tesque) for 1 h at room temperature. Primary antibodies were used against the following antigens: DCX (mouse, 1:500, Santa Cruz, Dallas TX, USA, sc‐271390), FOXA2 (goat, 1:500, R&D Systems, Minneapolis, MN, USA, AF2400), SOX2 (rabbit, 1:500, Millipore, Burlington, MA, USA, AB5602), TH (rabbit, 1:500, Millipore, AB152), and TH (mouse, 1:500, Millipore, MAB318). Primary antibody reactions were detected with either Alexa Fluor 488– or Alexa Fluor 555‐conjugated donkey secondary antibodies (1:500, Thermo Fisher Scientific). The cell nuclei were counterstained with Hoechst 33342 (1:4000, Dojindo, Kumamoto, Japan).

### 2.5 Polymerase chain reaction (PCR): Reverse transcription PCR (RT‐PCR) and quantitative PCR

Total RNA was extracted from the samples using NucleoSpin RNA (Macherey‐Nagel, Düren, Germany). Reverse transcription was performed with the High‐Capacity cDNA Reverse Transcription Kit (Thermo Fisher Scientific). PCR was performed with the KOD One PCR Master Mix (TOYOBO Co. Ltd., Osaka, Japan). Quantitative PCR was performed using the PowerUp SYBR Green Master Mix (Thermo Fisher Scientific) on an ABI7500 Real‐Time PCR System (Thermo Fisher Scientific). The data were obtained based on the ∆C_T_ method normalizing the raw data to the *GAPDH* gene. The primer sequences are listed in Table 1.

**TABLE 1:** PCR primers.

Target	Forward (5′‐3′)	Reverse (5′‐3′)	Product size (bp)
*CHRNA4*	TTCATCCGTGCTGTTGTGTG	ACAGGTGATGGACAAACTGC	115
*CHRNA6*	AGGCCTGTGGAAAACGTTTC	TGTGACGCAGCCACAAATTG	118
*CHRNA7*	TTTGGCTTGGCGAGATTTGG	AACCGTAAGCAACACGACTG	97
*FOXA2*	TTCAGGCCCGGCTAACTCT	AGTCTCGACCCCCACTTGCT	67
*GAPDH*	TTGAGGTCAATGAAGGGGTC	GAAGGTGAAGGTCGGAGTCA	117
*LMX1A*	GATCCCTTCCGACAGGGTCTC	GGTTTCCCACTCTGGACTGC	175
*LMO3*	CTCTCAGTCCAGCCAGACACCA	GGCACACTTCAGGCAGTCTTCA	120
*NR4A2*	CAGCTCCGATTTCTTAACTCCAG	GGTGAGGTCCATGCTAAACTTGA	52

### 2.6 Statistical analysis

All data are shown as the mean ± standard error of the mean (SEM). Statistical significance was determined by one‐way analysis of variance followed by Tukey's post hoc test. Statistical analyses were performed using Prism 9 (GraphPad, San Diego, CA, USA).

## 3 RESULTS

**FIGURE 1:**
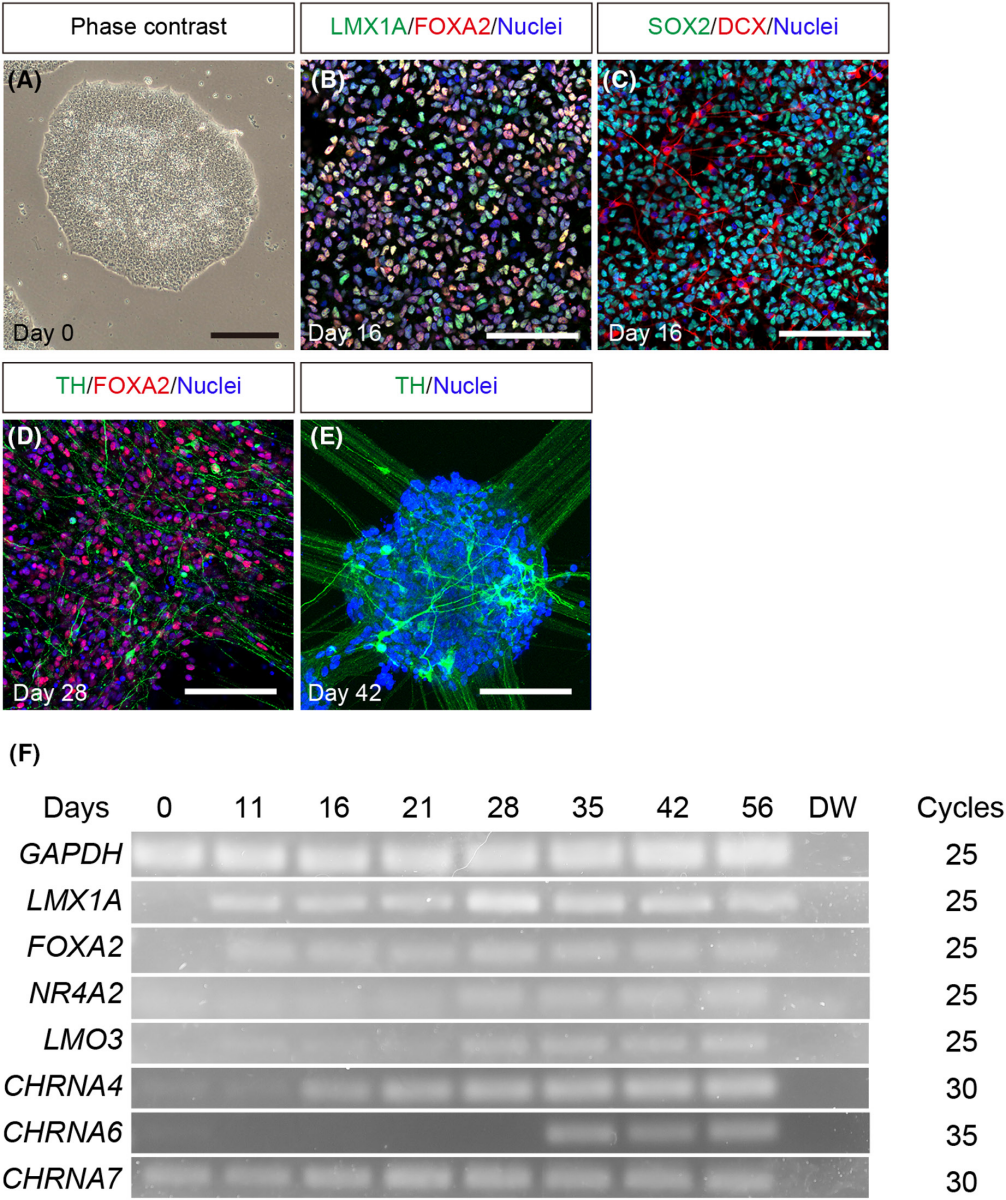
mDA neuron differentiation from hiPSCs and gene expression of nAChRs. (A) Phase contrast of undifferentiated hiPSCs. Expression of ventral midbrain markers, LMX1A and FOXA2, on day 16 (B). Expression of a neural progenitor marker, SOX2, and an immature neuron marker, DCX, on day 16 (C). Expression of a DA neuron marker, TH, and FOXA2 on day 28 (D) and TH on day 42 (E). Scale bars, 200 μm (A) and 100 μm (B–E). The reproducibility of the differentiation protocol by independent experiments is shown in Figure S1. (F) RT‐PCR analysis of midbrain TFs (*LMX1A*, *FOXA2*, *NR4A2*, and *LMO3*) and nAChRs (*CHRNA4*, *CHRNA6*, and *CHRNA7*) during mDA neuron differentiation from hiPSCs. *GAPDH* expression is shown as an internal control. Gel data is shown in Figure S2. DA, dopaminergic; hiPSCs, human induced pluripotent stem cells; mDA, midbrain dopaminergic; nAChRs, nicotinic acetylcholine receptors

Midbrain DA neurons were induced from hiPSCs using a simplified protocol based on a recently developed method.^22^ hiPSCs generated LMX1A^+^ and FOXA2^+^ mDA progenitors on day 16 (Figure 1A,B). At this stage, SOX2^+^ cells (neural progenitors) were more frequently observed than DCX^+^ cells (immature neurons) (Figure 1C), indicating that neural progenitors are indeed induced on day 16. Furthermore, TH^+^ neurons (DA neurons) were found on day 28 (Figure 1D) and went on to form colony‐like structures and elongated neurites on day 42 (Figure 1E).

Gene expression analysis via RT‐PCR revealed that the ventral midbrain progenitor transcription factors (TFs) *LMX1A* and *FOXA2* were expressed on day 11. The postmitotic ventral midbrain progenitor TF *NR4A2* (also known as *NURR1*) was subsequently expressed on day 28 (Figure 1F). Furthermore, the SNC TF *LMO3* was expressed on day 28 (Figure 1F). The representative midbrain TFs are sequentially expressed during mDA differentiation, mimicking the expression profile of mammalian ventral midbrain development.^19^, ^23^ These data indicate that our simplified protocol induced mDA neurons that closely resembled the process of ventral midbrain development.

We then investigated the expression of nAChR subtypes, *CHRNA4*, *CHRNA6*, and *CHRNA7*, during mDA neuron differentiation from hiPSCs by RT‐PCR (Figure 1F). *CHRNA7* was constitutively expressed across the entire differentiation process, including the undifferentiated stage (Figure 1F). *CHRNA4* was expressed on day 16, the mDA neuron precursor phase, and *CHRNA6* was expressed on day 35, the mDA neuron mature stage (Figure 1F). These results suggest that each nAChR subtype may have a unique function at each stage of mDA neuron differentiation.

**FIGURE 2:**
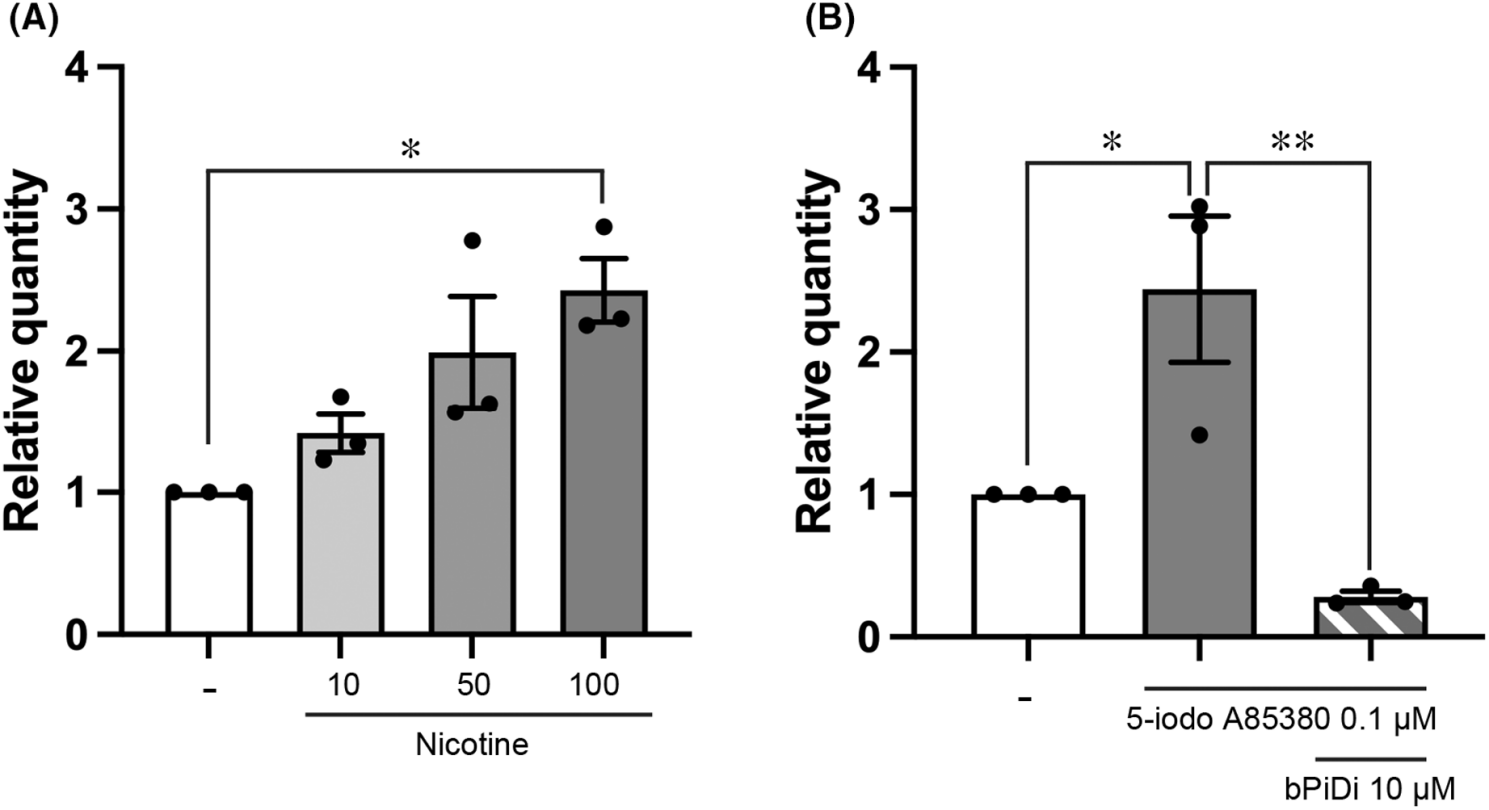
*LMO3* gene expression in hiPSC‐derived mDA neurons following nicotinic stimulation. (A) *LMO3* expression in hiPSC‐derived mDA neurons following treatment with nicotine (10–100 μM). (B) *LMO3* expression in hiPSC‐derived mDA neurons following treatment with 5‐iodo A85380 (0.1 μM), a selective α6 nAChR agonist, with or without bPiDi (10 μM), an α6 nAChR antagonist. Treatments were applied from day 28 to day 42 and differentiation was analyzed by quantitative PCR. Each value is represented by the mean ± SEM (*n* = 3). Significance (one‐way analysis of variance with Tukey's test): *p < 0.05, **p < 0.01. The raw data is shown in Table S1. hiPSCs, human induced pluripotent stem cells; mDA, midbrain dopaminergic; nAChRs, nicotinic acetylcholine receptors

To examine the role of nAChR in the maturation of mDA neurons, we focused on *CHRNA6*, which is expressed during the mDA neuron maturation phase. Mature mDA neurons (days 28–42) were treated with nicotine (10–100 μM, nonselective nAChRs agonist). The results showed that nicotine treatment significantly increased *LMO3* expression in a concentration‐dependent manner. (Figure 2A). Moreover, 5‐iodo A85380 (0.1 μM, a selective α6 nAChR agonist) also increased *LMO3* expression, and this increase was suppressed by simultaneous treatment with bPiDi (10 μM, a selective α6 nAChR antagonist) (Figure 2B).

## 4 DISCUSSION

To the best of our knowledge, this was the first study to characterize the expression pattern of the nAChR subunit genes *CHRNA4*, *CHRNA6*, and *CHRNA7* during DA neuron differentiation from hiPSCs. *CHRNA4* expression began on day 16, corresponding to the mDA neuron progenitor stage, whereas *CHRNA6* expression began on day 28, corresponding to the mature mDA neuronal stage. *CHRNA7* was expressed throughout the differentiation process, including in the undifferentiated hiPSCs. These results demonstrate the functional diversity of nAChRs in mDA differentiation from hiPSCs. Additionally, we found that the pharmacological stimulation of the α6 nAChR subunit in mature mDA neurons significantly enhanced *LMO3* expression, which is a gene expressed in SNC DA neurons in the embryonic and adult mammalian brain.^18^, ^19^, ^24^

LMO3 is a transcriptional coactivator of Pitx3, which is expressed in mammalian mDA neurons.^18^ LMO3 is predominantly expressed in the SNC, interacts with TFs, including Pitx3, and may be involved in SNC differentiation during midbrain development. In general, the LMO family is an important coregulator that interacts with TFs to acquire neuronal identity during brain development.^25^ Therefore, the increased *LMO3* expression may be involved in developing SNC DA neuron identity during hiPSC differentiation. Recent single‐cell transcriptomics revealed that hiPSC‐derived mDA neurons are divided into three subtypes: DAn1, DAn2, and DAn3.^24^ Gene expression analysis revealed that DAn1 and DAn2 neurons are the most mature DA subtypes, whereas DAn3 neurons expressed genes that are commonly associated with glutamatergic and GABAergic identities. The DAn1 subtype predominantly expressed *LMO3*, which was coexpressed with TH, a DA neuron marker. Similarly, human ventral midbrain scRNA‐seq data revealed that *LMO3* is specifically expressed in the DA2 population, for which the assumed DA neuron progenitor is the SNC. Additionally, *CHRNA6* is expressed in the DA2 population in the developing human ventral midbrain.^19^

This study used a simple method to promote maturation in hiPSC‐derived mDA neurons. Our data suggest that stimulating the α6 nAChR subunit on hiPSC‐derived mDA neurons may induce neuronal maturation that is biased toward SNC DA neurons.

## AUTHOR CONTRIBUTIONS

Takeshi Kato performed experiments and wrote the manuscript. Kaneyasu Nishimura designed the project, performed experiments, wrote the manuscript, and obtained a grant for the research. Masahiro Hirao performed experiments. Shun Shimohama supervised the project. Kazuyuki Takata designed the project, wrote the manuscript, and obtained grants for the research. All authors reviewed the manuscript.

## FUNDING INFORMATION

The study was supported by grants‐in‐aid from the Private University Research Branding Project of the Ministry of Education, Culture, Sports, Science, and Technology, the Japan Society for the Promotion of Science (JSPS) KAKEN (grant numbers 19K07854 and 22K07382 to KN, 20H03569 to KT), the Kobayashi Foundation (KT), the Shimizu Foundation for Immunology and Neuroscience (KT), the Smoking Research Foundation (KT), and Kyoto Pharmaceutical University Fund for Collaborative Research (KT).

## CONFLICT OF INTEREST STATEMENT

The authors declare no conflict of interest.

## ETHICS STATEMENT

Approval of the research protocols by an institutional reviewer board: This experiment was approved by the Ethical Review Committee for Medical and Health Research Involving Human Subjects at Kyoto Pharmaceutical University.

Informed Consent: Not applicable.

Registry and the registration No. of the study/trial: Not applicable.

Animal studies: Not applicable.

## Supporting information


Figure S1.

Table S1.

## Data Availability

The data that supports the findings of this study are available in the supplementary material of this article.
