# Supplementary material for: Expression and role of nicotinic acetylcholine receptors during midbrain dopaminergic neuron differentiation from human induced pluripotent stem cells
Source: Neuropsychopharmacol Rep. 2023 Jun 27;43(3):440–5. doi: 10.1002/npr2.12361 (PMC10496050; doi:10.1002/npr2.12361)
Supplement: Supplementary file 1 — Figure S1. [file NPR2-43-440-s001.pdf]

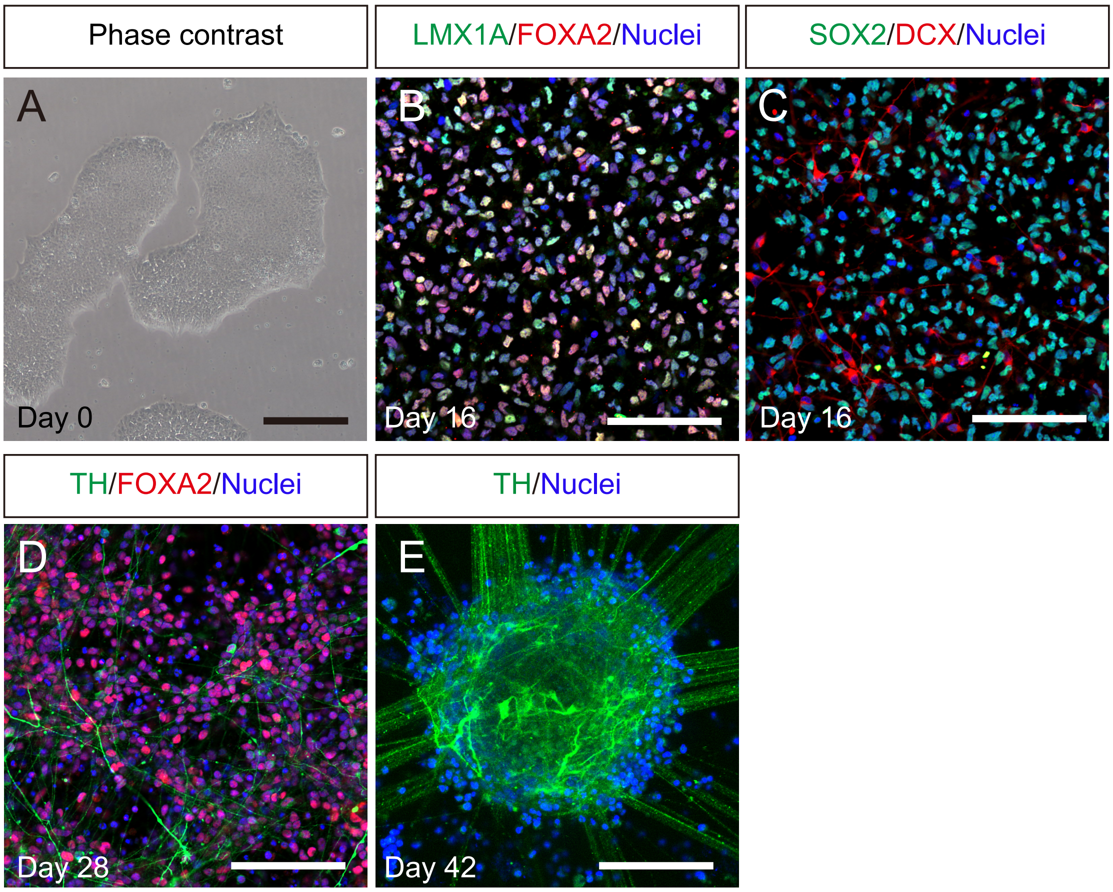

Supplementary Figure S1. Reproducibility of mDA neuron differentiation from hiPSCs in different batch of hiPSC-induction, related to Figure 1A-E. (**A**) Phase contrast of undifferentiated hiPSCs. Expression of LMX1A and FOXA2 on day 16 (**B**). Expression of SOX2 and DCX on day 16 (**C**). Expression of TH and FOXA2 on day 28 (**D**) and TH on day 42 (**E**). Scale bars, 200 µm (**A**) and 100 µm (**B**–**E**).

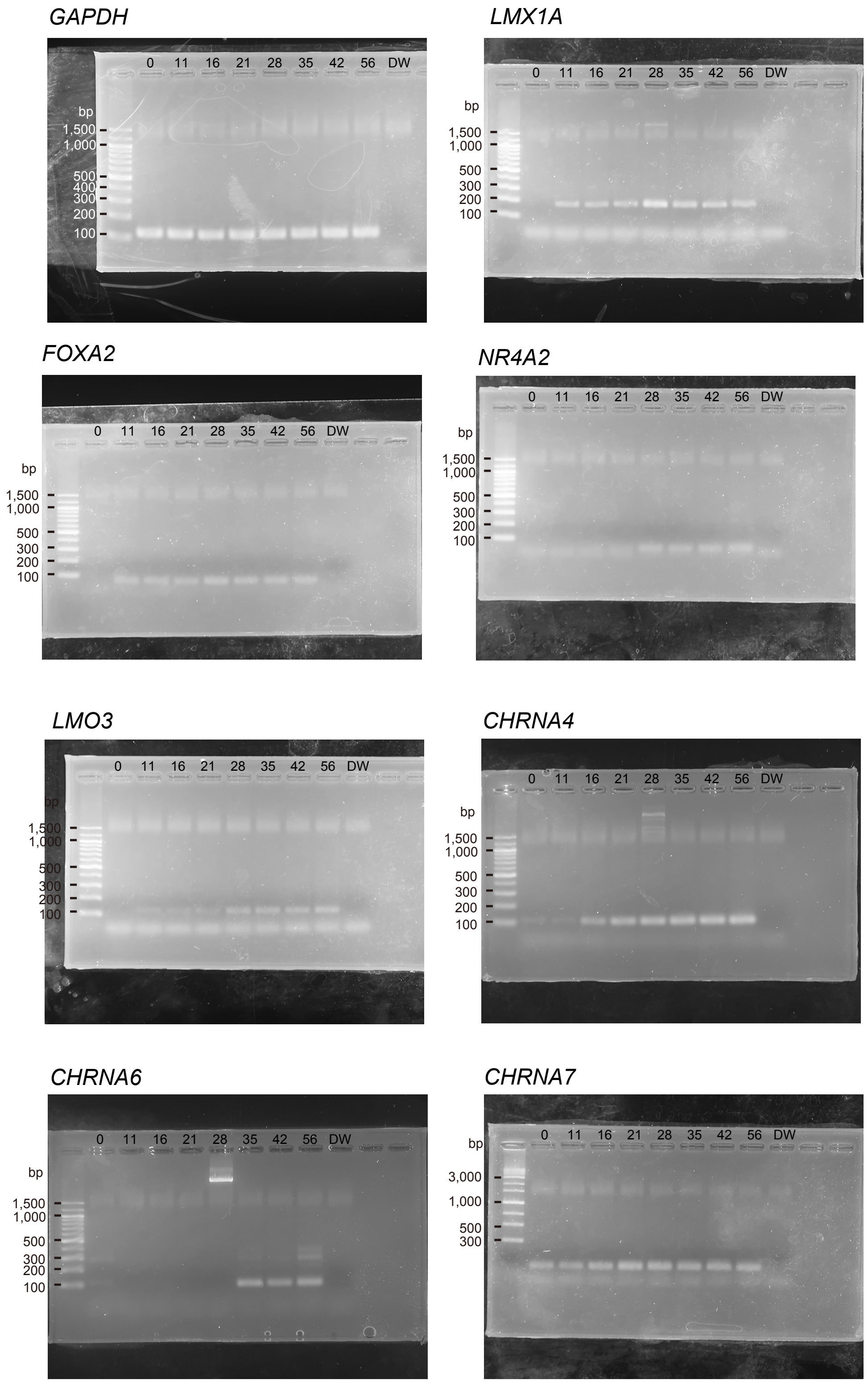

Supplementary Figure S2. Gel data of gene expression analysis during mDA neuron differentiation, related to Figure 1F.
